# microRNA-188 acts as a tumour suppressor in glioma by directly targeting the IGF2BP2 gene

**DOI:** 10.3892/mmr.2022.12826

**Published:** 2022-08-16

**Authors:** Li Ding, Ling Wang, Feng Guo

Mol Med Rep 16: 7124–7130, 2017; DOI: 10.3892/mmr.2017.7433

Following the publication of this paper, it was drawn to the Editors' attention by a concerned reader that cell migration assay data shown in Figs. 2 and 4 were strikingly similar to data that had appeared in different form in other articles by different authors (in addition to the apparent duplication of some of these data within this paper itself). Owing to the fact that the contentious data in the above article had already been published elsewhere, or were already under consideration for publication, prior to its submission to *Molecular Medicine Reports*, the Editor has decided that this paper should be retracted from the Journal. The authors were asked for an explanation to account for these concerns, but the Editorial Office did not receive a reply. The Editor apologizes to the readership for any inconvenience caused.

